# Percutaneous Aspiration, Lavage and Antibiotic Instillation. New Approach in the Management of Acute Calculous Cholecystitis

**DOI:** 10.1155/1991/78232

**Published:** 1991

**Authors:** Aws S. Salim

**Affiliations:** 1 University Department of Surgery at the Medical City in Baghdad Iraq; 2 The Department of Surgery Ward 6 Stobhill General Hospital Glasgow G21 3UW UK

## Abstract

Distension of the gallbladder and bacterial infection can perpetuate an attack of acute calculous cholecystitis and produce its local and systemic complications. This prospective randomized trial was conducted on patients with their first episode of acute calculous cholecystitis which was associated with pyrexia and tachycardia to examine whether ultrasound guided percutaneous aspiration and lavage of the gallbladder followed by intra-lumenal instillation of 500 mg ampicillin (PALA) enhanced recovery from cholecystitis. Twenty patients were randomized to receive 500 mg of ampicillin every 6 hours for 5 days and another 20 patients were randomized to receive this treatment in addition to PALA within 12 hours of admission. Twenty four hours after admission to hospital, all the patients treated with PALA were apyrexial and had no residual right hypochondrial tenderness or guarding, a result superior (p < 0.001) to that of the group without PALA where at least 75% of patients were still showing these signs. Two days after admission the WBC count of the PALA group was significantly (p < 0.05) lower than that of the other group (6.32 ± 0.1 × 10^9^/L vs 10.31 ± 0.4 × 10^9^/L, mean ± SEM, n = 20). Four days after admission, all members of the PALA group were comfortably tolerating solid food for the previous 24 hours and were, therefore, discharged home whereas all members of the other group were still in hospital and 85% of them were discharged home after hospitalization for 6 to 7 days. Three members (15%) of this group deteriorated and underwent emergency surgery.

The results show that addition of PALA to the conventional non-operative treatment of acute cholecystitis enhances recovery and avoids the complications necessitating emergency surgery.


HPB Surgery, 1991, Vol. 3, pp. 167-176
Reprints available directly from the publisher
Photocopying permitted by license only
© 1991 Harwood Academic Publishers GmbH
Printed in the United Kingdom

### PERCUTANEOUS ASPIRATION, LAVAGE AND ANTIBIOTIC INSTILLATION. NEW APPROACH IN THE MANAGEMENT OF ACUTE CALCULOUS CHOLECYSTITIS

AWS S. SALIM
*University Department of Surgery at the Medical City in Baghdad, Iraq*

*(Received 5 March 1990, in final form 7 May 1990)*

Distension of the gallbladder and bacterial infection can perpetuate an attack of acute calculous cholecystitis and produce its local and systemic complications. This prospective randomized trial was conducted on patients with their first episode of acute calculous cholecystitis which was associated with pyrexia and tachycardia to examine whether ultrasound guided percutaneous aspiration and lavage of the gallbladder followed by intra-lumenal instillation of 500 mg ampicillin (PALA) enhanced recovery from cholecystitis. Twenty patients were randomized to receive 500 mg of ampicillin every 6 hours for 5 days and another 20 patients were randomized to receive this treatment in addition to PALA within 12 hours of admission. Twenty four hours after admission to hospital, all the patients treated with PALA were apyrexial and had no residual right hypochondrial tenderness or guarding, a result superior ($p < 0.001$) to that of the group without PALA where at least 75% of patients were still showing these signs. Two days after admission the WBC count of the PALA group was significantly ($p < 0.05$) lower than that of the other group ($6.32 \pm 0.1 \times 10^9$/L vs $10.31 \pm 0.4 \times 10^9$/L, mean ± SEM, $n = 20$). Four days after admission, all members of the PALA group were comfortably tolerating solid food for the previous 24 hours and were, therefore, discharged home whereas all members of the other group were still in hospital and 85% of them were discharged home after hospitalization for 6 to 7 days. Three members (15%) of this group deteriorated and underwent emergency surgery.

The results show that addition of PALA to the conventional non-operative treatment of acute cholecystitis enhances recovery and avoids the complications necessitating emergency surgery.

KEY WORDS: percutaneous, antibiotic, ampicillin, calculous, cholecystitis

### INTRODUCTION

Although there is no dispute that in an otherwise healthy subject cholecystectomy is a safe procedure for the treatment of symptomatic gallstones, the timing of this operation remains controversial[1-4]. Acute cholecystitis is the most frequent complication of gallstones and may progress to a mucocele, "empyema", perforation, gangrene, fistula formation or ascending cholangitis[5-7]. Consequently, emergency surgery may be necessary for some cases, however, its mortality and morbidity rates continue to be higher than those of elective surgery[3,4].

From the pathological features and natural history of acute cholecystitis it can be reasoned that most attacks will remit, thus enabling emergency surgery to be

Correspondence and reprint requests to: Aws. S. Salim, The Department of Surgery, Ward 6, Stobhill General Hospital, Glasgow G21 3UW, U.K.

avoided[3-7]. It follows that any measures aimed at improving the outcome of non-operative management of acute cholecystitis may play a positive role in reducing the risks of this disorder. Infection and increased tension within the gallbladder causing a diminished vascular supply are the major factors responsible for producing the complications that can be associated with an attack of acute cholecystitis. Theoretically, the antibiotic attaining the highest concentration in the biliary tree and being most active against the commonest organisms infecting it would be chosen for conservative treatment of cholecystitis[6]. Much has been written on the concentration which various antibiotics can attain in the biliary system[8], but if there is a blockage of the cystic or the common bile ducts with superadded infection, this property becomes of doubtful value[6]. The present study was, therefore designed to determine whether percutaneous aspiration of the gallbladder followed by lavage and instillation of an antibiotic improves the outcome of conservative treatment of acute cholecystitis.

### PATIENTS AND METHODS

### *Patients*

This is a prospective randomized trial conducted on patients with their first attack of acute calculous cholecystitis which was associated with pyrexia and tachycardia. Randomization was by drawing sealed envelopes. Treatment started immediately after the diagnosis was made.

Patients judged suitable for this study were those presenting with clinical signs and symptoms (including a positive Murphy's sign) of acute cholecystitis coupled with pyrexia (more than 38°C), tachycardia, leucocytosis, normal serum amylase levels and liver function tests, and ultrasonic evidence of a distended gallbladder containing one or more stones.

### *Exclusion Criteria*

Patients were not recruited into the study if there was evidence of one or more of the following: septic shock, jaundice, diabetes, pregnancy, alcoholism, sensitivity to penicillins, hepatic or renal disorders, serious underlying disease — for example cardiorespiratory problems, or other gastrointestinal disorders like reflux oesophagitis, hiatus herniation, peptic ulceration, the Zollinger-Ellison syndrome, Crohn's disease, ulcerative colitis, or irritable bowel syndrome. These conditions would make it difficult to assess the significance of the patient's signs and symptoms. History of biliary disease, previous gastrointestinal surgery, or concomitant treatment with any medication made the patient unsuitable for this study. In addition, patients were not studied if ultrasonically the gallbladder was not distended or contained no stones and if there was any suggestion of choledocholithiasis or dilatation of the biliary tree.

*The Study Design*

Acute cholecystitis was initially diagnosed from the history and physical examination. Patients were then completely fasted, given 50 mg of pethidine by intramuscular injection and admitted to hospital for bed rest and further management. In all cases radiological examination with plain abdominal films was undertaken to exclude visceral perforation. Standard haematology and biochemistry measurements (haemoglobin, erythrocyte sedimentation rate, packed cell volume, white blood cell count (WBC), electrolytes, urea, creatinine, urate, liver function tests) and urine examination were carried out.

After admission to hospital, patients were confined to bed until they were apyrexial and hydrated intraveneously. They were allowed nil orally for 24 hours then sips of water every hour for 12 hours followed by another 12 hours of no more than 100 mls of water every hour. If these volumes were well tolerated, the intravenous line was disconnected at the end of the last 12 hours and patients were allowed free fluid intake by mouth and gradually reintroduced to solid food. The intravenous maintenance fluids for hydration were one litre of dextrose-saline with 20 mmol potassium every 8 hours. Analgesia was provided by intramuscular pethidine (not exceeding 50 mg every 4 hours). In all cases, ampicillin B.P. (Penbritin, Beecham Laboratories, Brentford, U.K.), 500 mg every 6 hours, was given by intramuscular injection for 48 hours then, if possible, orally for 3 days (5 days of antibiotic treatment). Within 12 hours of admission patients were examined ultrasonically and once this scan confirmed the diagnosis of acute calculous cholecystitis, they were randomized to have ultrasound-guided percutaneous aspiration of the gallbladder with lavage and instillation of ampicillin or to remain on their current therapeutic regimen. The percutaneous procedure was performed under ultrasound guidance as follows: with the patient in the left lateral position, a skin port of entry that enabled the gallbladder to be punctured directly was chosen just below the right ninth costal cartilage and the site was infiltrated with 2–3 ml of local anaesthetic (1% plain xylocaine). A long 20-guage needle (Cook Inc., Bloomington, Indiana, USA) was advanced directly into the lumen of the gallbladder via the fundus and its contents were aspirated until it was no longer distended ultrasonically. The aspirate was sent for a Gram stain and bacteriological culture (both aerobic and anaerobic). Five ml of double distilled water were instilled into the gallbladder and the same volume aspirated and discarded. This lavage was repeated then 500 mg of ampicillin powder in 5 ml double distilled water were instilled into the gallbladder. The needle was withdrawn and a small dressing applied over its port of entry.

The diagnostic ultrasonography was repeated 48 hours later to re-examine the size of the gallbladder. The WBC count was determined on each of the first three days after admission.

Progress was assessed by following the physical signs and symptoms (pain, fever, tenderness, guarding), the WBC count, the ultrasonic image of the gallbladder, and the return to and tolerance of solid food.

After recovery from the attack (that is, when all the signs and symptoms of acute cholecystitis had ceased, the WBC count had returned to normal and solid food had been tolerated without complications for at least 24 hours) patients were discharged home to be re-admitted for an elective cholecystectomy as soon as time was available on a routine operating list.

The end points of this study were successful recovery from the acute attack or failure of either of the non-operative policies employed.

### *Ethical Considerations*

This study was approved by the Ethical Committee of Human Experimentation of the hospital and every patient gave written informed consent.

### *Statistical Analysis*

Results are expressed as the mean ± SEM unless stated otherwise. The Wilcoxon's signed-ranks test for paired non-parametric data was used to determine the statistical significance ($p < 0.05$) of observed differences within each group and the Mann-Whitney U test for non-parametric data or the $X^2$ test with Yates' correction were used for comparisons between groups.

### RESULTS

The patients studied and treatment results are presented in Tables I, II.

### *Patient Characteristics*

Twenty patients were randomized to each of the study groups and their age and sex characteristics were well matched (Table 1).

**Table 1** Patient Characteristics

| | *Percutaneous treatment* | *No percutaneous treatment* |
|---|---|---|
| n | 20 | 20 |
| Age | | |
| mean year | 29 | 33 |
| range | 23–47 | 27–51 |
| Women (n) | 15 | 16 |
| Men (n) | 5 | 4 |

### *General Observations*

The duration of symptoms before admission to hospital was less than 24 hours in every member of the study groups. In the group treated percutaneously, 3 men and 5 women were smokers, and in the group without this treatment, 2 men and 4 women, were smokers. In the latter group, all the men ($n = 4$) and 2 women were social drinkers and in the group treated percutaneously, 4 men and 3 women were social drinkers. Three women in each group were nulliparous and all the women in the study had at some stage in their life used the contraceptive pill. At the time of admission, 5 women in the group treated percutaneously and 7 women in the other group were on this pill. None of the patients studied were hyperlipaemic or hypercholesterolaemic. Nine patients (45%) in the group treated percutaneously and 7 patients (35%) in the other group had a family history of gallstones.

Ultrasonically, 5 patients in the group treated conventionally and 7 patients in the group treated percutaneously had solitary gallbladder calculi, which in 2 members of each group appeared to be larger than one cm in diameter, while all the remaining patients had multiple calculi.

*The Percutaneous Procedure*

In all cases this procedure was very well tolerated and produced no pain or discomfort. None of the patients developed infection at the site of needle entry and the repeat ultrasound scan showed no evidence of right hypochondrial collection (gallbladder leakage) in any case.

*Comparison between the Groups (Table 2)*

The percutaneous treatment enhanced recovery from the acute attack and prevented patient deterioration. Soon after this treatment and within 12 hours of admission, 85% of the patients were relieved of their right hypochondrial pain and none had this pain 24 hours after admission. At corresponding time periods, the number of patients without this treatment who had pain was significantly ($p < 0.001$) higher and 48 hours after admission 15% of them were still having pain. It follows that the duration of analgesic treatment was significantly ($p < 0.001$) reduced by the percutaneous procedure. This procedure was within 12 hours after admission more effective than expectant treatment alone ($p < 0.01$) in reducing the number of patients having fever, tachycardia, a positive Murphy's sign, and right hypochondrial tenderness with or without guarding or rigidity. By 24 hours after admission, this effectiveness was further increased and none of the patients remained with any of these signs compared with at least 75% of patients given expectant treatment alone, ($p < 0.001$). Even 48 hours after admission, at least 20% of patients in the latter group were still symptomatic. The percutaneous procedure was more effective than traditional expectant treatment in controlling the magnitude of the inflammatory process produced by acute cholecystitis. At each of 24 and 48 hours after admission, the WBC count was significantly ($p < 0.05$) less than the previous 24 hours' count and by the second day after admission this count was significantly ($p < 0.05$) less than that of the group treated traditionally.

Although at diagnostic ultrasonography all gallbladders were distended, a further scan 48 hours after admission showed that none treated percutaneously remained distended compared with 20% of cases in the other group which remained distended. Bacteriological examination of the bile aspirated percutaneously showed no growth in 8 cases (40%) whilst the remaining cases predominantly grew one of Escherichia coli (40%), Klebsiella pneumoniae (15%) or Streptococcus faecalis (5%).

All the patients treated percutaneously comfortably tolerated free fluids by mouth 48 hours after admission to hospital and, therefore, their intravenous lines were disconnected making them freely mobile and unrestricted. Such progress was only observed in 60% of the patients in the other group ($p < 0.01$). Similarly, 72

**Table 2** Treatment of Acute Cholecystitis

| | *Percutaneous treatment* | *No percutaneous treatment* |
|---|---|---|
| Fever/tachycardia (n) | 20 (100%) | 20 (100%) |
| on admission | | |
| after 12 hrs | 12 (60%) | 20 (100%) |
| after 24 hrs | 0 | 17 (85%) |
| after 48 hrs | — | 6 (30%) |
| Rt hypochondrial pain (n) | | |
| on admission | 20 (100%) | 20 (100%) |
| after 12 hrs | 3 (15%) | 16 (80%) |
| after 24 hrs | 0 | 9 (45%) |
| after 48 hrs | — | 3 (15%) |
| + ve Murphy's sign (n) | | |
| on admission | 20 (100%) | 20 (100%) |
| after 12 hrs | 4 (20%) | 20 (100%) |
| after 24 hrs | 0 | 15 (75%) |
| after 48hrs | — | 4 (20%) |
| WBC ($\times 10^9$/L)—mean | | |
| on admission | $16.12 \pm 0.3$ | $14.11 \pm 0.5$ |
| after 24 hrs | $11.22 \pm 0.2$ | $13.21 \pm 0.6$ |
| after 48 hrs | $6.32 \pm 0.1$ | $10.31 \pm 0.4$ |
| after 72 hrs | $5.47 \pm 0.2$ | $6.11 \pm 0.5$ |
| Ultrasonically distended gallbladder (n) 48 hrs after admission | 0 | 4 (20%) |
| Patients deteriorating (n) | 0 | 3 (15%) |
| Patients tolerating free fluids by mouth 48 hrs after admission (n) | 20 (100%) | 12 (60%) |
| Patients tolerating solid food 72 hrs after admission (n) | 20 (100%) | 11 (55%) |
| Hospital stay (n) | | |
| 4 days | 20 (100%) | — |
| 6 days | — | 14 (70%) |
| 7 days | — | 3 (15%) |
| longer | — | 3 (15%) |

hours after admission, the number of patients in the latter group who were able to tolerate solid food for 24 hours was significantly ($p < 0.01$) less than that of the percutaneous group (100%). Consequently, all patients in this group were discharged home by the fourth day after admission while all the patients in the other group were still in hospital at this time period.

Three patients (15%) in the group treated conventionally did not respond to treatment and began to deteriorate. Therefore, 48 to 72 hours after admission they had emergency surgery during which no stones were found beyond the gallbladder.

All the patients who made a complete recovery from their acute attack remained well, without any complaints of digestive disturbances, biliary colics or attacks of fever, until they had an elective cholecystectomy some 6 weeks after they were discharged from hospital. During this operation, 2 patients in each of the study groups had stones in the common bile duct (missed by the earlier ultrasound scan).

### DISCUSSION

Acute cholecystitis is produced by obstruction of the cystic duct. The gallbladder wall becomes inflamed due to the irritation by the concentrated bile within it producing a chemical cholecystitis, but bacterial infection soon supervenes[5,6,7]. In most patients the condition will subside. However, in some cases there is the possibility of increased tension causing a diminished vascular supply which, in turn, leads to tension gangrene and perforation beginning at the fundus[3,4]. Short of this event, a gallbladder full of pus causes continued local and systemic disturbance[3,4]. It follows that reducing the tension within the gallbladder and eradicating infection should be the top priorities of non-operative management of acute cholecystitis. It is customary for this management to include an antibiotic when there is evidence that bacterial infection is important (high fever, local tenderness), however, obstruction of the biliary tree impairs its delivery into the lumen. Therefore, to enable successful treatment of acute cholecystitis and to avoid its complications, the present study introduced ultrasound guided percutaneous aspiration of the gallbladder followed by lavage and direct instillation of an antibiotic into its lumen. This procedure enhanced control of the inflammatory process produced by the acute cholecystitis, thus enabling patients to recover from the attack and to be discharged home after a significantly shorter time period than that after conventional treatment alone (Table 2). More importantly, the percutaneous procedure prevented failure of conventional treatment and the necessity of emergency surgery (Table 2).

Because an acute attack of cholecystitis may not resolve and may produce local and systemic complications, some advocate cholecystectomy as the sole management[3,4,6]. However, the morbidity and mortality of this approach remain higher than those of elective cholecystectomy[3,4]. Furthermore, a high level of expertise is required by the surgeon and there remains a group of patients who are at risk of developing wound sepsis, such as those over 50 years of age, those with jaundice, the obese and those who have been shown to have a non-functioning gallbladder[4,5]. If emergency surgery is not the favoured policy for the management of an acute attack and these cases are treated conservatively then the ones that fail to respond or develop complications should undergo the safer option of cholecystostomy[3], this last procedure is still emergency surgery which carries the risk of wound sepsis and demands longer hospital stay with all its implications on cost and manpower. Considering these pros and cons, the best management policy for acute cholecystitis would be to treat the attacks conservatively and to perform elective surgery at a later day, preferably within a few weeks after the acute attack has subsided. Addition of the percutaneous procedure presented in the present study would ensure success of this policy. It might be argued, however, that the procedure is invasive and may increase management costs. Such an argument cannot be sustained in view of the following observations. First, no adverse events were incurred by the percutaneous method and in particular it produced no discomfort, pain, sepsis or leakage. Second, the hospital stay of the patients treated percutaneously was less than that of those treated conventionally. Third, none of the percutaneous cases succumbed to the acute attack whereas 15% of the patients treated conventionally deteriorated and required emergency surgery. The success of the percutaneous procedure did not appear to be dependent on the number or size of calculi in the gallbladder, since these stone characteristics were roughly similar between the two study groups.

The use of ampicillin for the present study might be criticised. This antibiotic is a broad spectrum one which is active against the organisms commonly implicated in acute cholecystitis, it is well excreted in the bile even when the biliary tree is obstructed, and can be given orally or parenterally[6]. For these reasons it was employed in the present investigation, however, the use of antibiotic combinations might be even better in terms of the effectiveness in controlling the infection produced by cholecystitis. This last point would be particularly true if such combinations were to include an antibiotic effective against anaerobic organisms.

The percutaneous procedure was carried out using a needle. A significant advantage can be attained if after the needle has punctured the gallbladder, a plastic cannula was advanced into the gallbladder lumen and the needle removed. This would enable placement of the cannula deep into the gallbladder and complete evacuation of its contents without the fear of slipping out, thus achieving a more complete removal of septic material from the gallbladder and perhaps enabling any stone obstructing its outlet to slip back into the lumen. Furthermore, the volume of the material recovered can give an estimate of the best volume of distilled water for lavaging the gallbladder without causing distension.

In conclusion, this is the first report demonstrating that ultrasound guided aspiration of the gallbladder followed by lavage and direct instillation of an antibiotic into its lumen enhances control of acute attacks of cholecystitis afforded by conventional conservative management.

### *Acknowledgements*

I am very grateful to Professor A.M. Shukri for giving me the opportunity to undertake this work in his department. I thank the Surgical Residents and nursing staff at this department for their help and support during the course of the investigation. The skilful secretarial work of Mrs. Moira Cairney is gratefully acknowledged.

### *References*

1. Lund, J. (1960) Surgical indications in cholelithiasis: prophylactic cholecystectomy elucidated on the basis of long-term follow up on 526 nonoperated cases. *Ann. Surg.* **151**, 153–162
2. Way, L.W. Admirand, W.H. and Dunphy, J.E. (1972) Management of choledocholithiasis. *Ann. Surg.* **176**, 347–359
3. Dudley, H.A.F. (1986) The biliary tract. In: *Hamilton Bailey's Emergency Surgery*. Edited by H.A.F. Dudley. 11th Edition, pp. 312–321. Bristol: Wright
4. Davidson, C.M. (1987) The biliary system. In: *Operative Surgery and Management*. Edited by G. Keen. 2nd Edition, pp. 272–290. Bristol: Wright
5. Haw, C.S. and Gunn, A.A. (1973) The significance of infection in biliary disease. *J. Roy. Coll. Surg. Ed.* **18**, 209–212
6. Smiddy, F.G. (1976) The complications of cholelithiasis. In: *The Medical Management of the Surgical Patient*, pp. 28–30. London: Edward Arnold

7. Keighley, M.R.B. (1982) Infection and the biliary tree. In: *Clinical Surgery International* "The Biliary Tract". Edited by L.H. Blumgart. pp. 219–235. London: Churchill Livingston
8. Berger, S.A., Levy, Y., Halevy, A., Gorea, A. and Orda, R. (1988) Penetration of cefazolin, ceftriaxone, cefoperazone, and ceftazidime into human gallbladder tissue and bile. *World J. Surg.* **12**, 641–644

*(Accepted by S. Bengmark 7 May 1990)*

### INVITED COMMENTARY:

We commend Dr. Salim for a very interesting and well-performed study. Clearly these data demonstrate that percutaneous gallbladder evacuation with antibiotic instillation is superior to parenteral antibiotics alone for the nonoperative treatment of acute cholecystitis. Realistically, at this time we cannot advocate performing percutaneous gallbladder lavage and antibiotic instillation on all patients presenting with acute cholecystitis for two reasons: 1. The treatment involves an invasive procedure which, though well tolerated in the present study, will be associated with some degree of technical complications and morbidity. 2. The authors are recommending that this procedure be performed in a group of patients in which noninvasive therapy, parenteral antibiotics alone, will be successful for most. More appropriately, percutaneous gallbladder lavage and antibiotic instillation should be reserved for patients presenting with uncomplicated acute cholecystitis who fail to show improvement with 12 to 24 hours of parenteral antibiotics.

More importantly, these data lend further impetus to the developing field of nonoperative therapy for calculus disease of the gallbladder. There is a growing body of literature demonstrating the success of biliary lithotripsy in combination with oral bile acid therapy for the treatment of cholelithiasis. However, with the current clinical restrictions, only approximately 30% of patients presenting with cholelithiasis will be eligible for this treatment. In addition, early studies with biliary lithotripsy and oral bile acid therapy have suggested a 10% per year recurrence rate. For these reasons, other treatment modalities must be developed if nonoperative therapy of calculus disease of the gallbladder is to become a reality. As Dr. Salim has mentioned in his discussion, percutaneous cholecystostomy is one such important treatment modality. As of 1989, only 231 cases of percutaneous cholecystostomy had been reported in the English language literature. These studies generally were comprised of patients with advanced systemic illnesses, such as malignancy, severe cardiorespiratory disease, and sepsis with organ failure. Following percutaneous cholecystostomy, symptoms of cholecystitis improved or resolved in approximately 90% of these high-risk patients within 24–48 hours. Furthermore, when these patients stabilized, many were then able to tolerate definitive cholecystectomy or simply had their drains removed without recurrent symptoms. The overall complication rate cited in the literature is 8%, and there have been only 2 deaths attributed to the procedure. Therefore, percutaneous cholecystostomy appears to be a relatively safe and effective alternative in the treatment of cholecystitis. In addition, percutaneous cholecystostomy also provides a portal by which gallbladder calculi can be extracted, dissolved, and/or fragmented. Currently, the most promising gallstone solvent under development is methyl-tert-butyl-ether (MTBE). In animal models and a few selected patients, percutaneous cholecystostomy coupled with MTBE infusion has been effective in dissolving cholesterol gallstones. The further development of percutaneous cholecystostomy and gallstone dissolution is critical since all patients with radiolucent gallstones will be eligible for this therapy, regardless of the number and size of their stones. However, as with lithotripsy, gallstone dissolution leaves the gallbladder *in situ* and therefore is associated with a risk of recurrent stone formation. To combat this problem, there have been experimental attempts at percutaneous gallbladder ablation. Through a percutaneous cholecystostomy tube, the cystic duct is occluded by a balloon catheter or plugged with a substance such as cyanoacrylinitrocellulose. Following this, a noxious agent such as absolute ethanol is instilled into the gallbladder resulting in mucosal necrosis and mural fibrosis.

We believe the future of gallstone therapy will involve nonoperative percutaneous procedures such as those we have just described. The data provided by Dr. Salim clearly illustrate that percutaneous evacuation of the gallbladder in the setting of acute cholecystitis, results in a rapid resolution of the inflammatory process. We envisage the day when percutaneous cholecystostomy with stone extraction or dissolution, followed by gallbladder ablation, will be the procedure of choice for the treatment of calculus disease of the gallbladder.

Professor Richard L. Simmons
Department of Surgery
University of Pittsburgh
1084 Scaife Hall
PITTSBURGH, Pennsylvania 15261
USA

